# GLOBAL POPULATION AGING AND HEAT EXPOSURE IN THE 21ST CENTURY: IMPLICATIONS FOR LATE-LIFE WELL-BEING AND POLICY

**DOI:** 10.1093/geroni/igad104.0499

**Published:** 2023-12-21

**Authors:** Deborah Carr, Giacomo Falchetta, Ian Sue Wing, Enrica DeCian

**Affiliations:** Boston University, Boston, Massachusetts, United States; International Institute for Applied Systems Analysis, Laxenberg, Salzburg, Austria; Boston University, Boston, Massachusetts, United States; Ca’ Foscari University of Venice, Venice, Veneto, Italy

## Abstract

Climate change has profound consequences for older adults’ well-being. Global increases in the frequency, intensity and duration of extreme heat spells pose the most direct threats, given older adults’ underlying health conditions and reduced capacity to thermoregulate. Individual-level consequences of heat exposure are well-documented, yet gerontological research has paid less attention to older adults’ heat exposure at the population level. World regions with both increasing concentrations of older adults and high temperature extremes are potential hotspots where growing aged populations are at risk of heat-related health threats. We estimate older adults’ current and future heat exposure in six global regions (Africa, Asia, Europe, North America, Oceania, South America). Analyses use NASA NEX Global Daily Downscaled Product climate data and country-level projections for the size and distribution of the age 65+ population. We focus on measures of cumulative heat exposure (cooling degree days) and acute exposure to heat extremes (95th percentile of daily maximum temperatures). We project that by 2050, populations aged 65+ globally exposed to >1200 cooling degree days will double. We also project that one-quarter of the aged population worldwide (roughly 340 million) will be living in climates with 95th percentile TMax95 > 99.5 F◦, a critical exposure threshold for health. The drivers of this population-level exposure vary by region. Population aging will drive exposure in historically hot regions in Africa and Asia, whereas climate change will drive heat exposure in historically older regions in Europe and North America. These results can inform public policy and local decision-making.

